# Complications of minimally invasive surgery for primary open-angle glaucoma in patients with diabetic retinopathy: a retrospective cohort study

**DOI:** 10.1007/s10792-026-03945-8

**Published:** 2026-02-05

**Authors:** Rishith Vaddavalli, Nada Madkour, Jessan A. Jishu, Mohammad H. Hussein, Ahmed A. Abdelghany, Ahmed Abdelmaksoud, Manal S. Fawzy, Eman A. Toraih

**Affiliations:** 1 Tulane University School of Medicine, New Orleans, LA 70112 USA; 2 Faculty of Medicine, Alexandria University, Alexandria, Egypt; 3 Ochsner Clinic Foundation, New Orleans, LA 70112 USA; 4 Department of Ophthalmology, Faculty of Medicine, Suez Canal University, Ismailia, 41522 Egypt; 5 Department of Internal Medicine, University of California, Riverside, 92521 USA; 6 Center for Health Research, Northern Border University, 73213 Arar, Saudi Arabia; 7 Department of Surgery, Tulane University School of Medicine, New Orleans, LA 70112 USA; 8 Department of Cardiovascular Perfusion, SUNY Upstate Medical University, New York, NY 13210 USA; 9 Genetics Unit, Department of Histology and Cell Biology, Faculty of Medicine, Suez Canal University, Ismailia, 41522 Egypt

**Keywords:** Minimally invasive glaucoma surgery, Primary open-angle glaucoma, Diabetic retinopathy, Vision loss, Ocular hemorrhage, TriNetX

## Abstract

**Objective:**

Minimally invasive glaucoma surgery (MIGS) has become increasingly popular for treating primary open-angle glaucoma (POAG). However, data regarding complications for patients with comorbid diabetic retinopathy (DR) are limited. This study aimed to compare complications after MIGS in POAG patients with and without DR.

**Methods:**

This is a retrospective cohort study using the TriNetX global health network. Adult patients with POAG who underwent MIGS were identified, with one group having comorbid DR and the other without. Propensity score matching was applied, yielding 518 patients per group for analysis. Complications assessed included vision loss, hypotony, ocular hypertension, cataract formation, eye infection, and any ocular hemorrhage. The primary outcomes were post-procedure complication rates and their statistical significance. Hazard ratios (HRs) with 95% confidence intervals (CIs) were computed.

**Results:**

Patients with DR who underwent MIGS had a higher risk for vision loss (32.8% vs. 24.4%, HR 1.443, 95% CI 1.13–1.841) and ocular hemorrhage (13.4% vs. 4.3%, HR 3.194, 95% CI 1.929–5.288) compared to those without DR. Cataract formation rates were lower in DR patients at 3 months (44.4% vs. 50.6%, *p* = 0.046) and 6 months (53.3% vs. 59.5%, *p* = 0.048) post-surgery. No significant differences were observed in rates of hypotony, ocular hypertension, or eye infection.

**Conclusion:**

MIGS in patients with DR is associated with an increased risk of post-procedure complications, particularly vision loss and ocular hemorrhage. These findings can aid in the clinical management and counseling of patients with both POAG and DR considering MIGS.

**Supplementary Information:**

The online version contains supplementary material available at 10.1007/s10792-026-03945-8.

## Introduction

Glaucoma, a group of progressive optic neuropathies, leads to the degeneration of retinal ganglion cells and worsening visual loss [1]. The most common subtype is primary open-angle glaucoma (POAG), which carries a global prevalence of almost 70% of the total glaucoma cases, or 69 million in the adult population [2]. Except for normal-pressure glaucoma, this subtype of glaucoma is characterized by diminished aqueous outflow, which leads to fluid accumulation, elevated intraocular pressure, and gradual optic nerve damage [3].

There are several risk factors for this, with diabetes mellitus (DM) being a primary and prevalent one. Indeed, case–control studies have shown that patients with DM have approximately a 49% increased odds of developing POAG compared with those without DM [4, 5]. This correlation exists because chronic hyperglycemia impairs regulation of retinal blood flow and hypoxic responses, and the constant remodeling of connective tissue at the head of the optic nerve, two mechanisms that can increase mechanical stress and fibrosis [6]. This can exacerbate resistance to aqueous humor outflow, subsequently increasing intraocular pressure [7]. Such pathophysiology is specifically explained by the occurrence of diabetic retinopathy (DR), an ocular complication of DM characterized by progressive retinal vascular damage that leads to vision-threatening sequelae, including microaneurysms, hemorrhage, vessel leakage, and ischemia [8, 9].

Currently, the only proven treatment for POAG is reducing intraocular pressure through medical or procedural interventions [10]. Topical medications with prostaglandins, cholinergic agonists, and beta-blockers are often used as initial therapies. Still, they can result in low adherence if used long-term, especially for asymptomatic conditions, and come with adverse ocular and cardiovascular effects [11]. Trabeculectomy has been the most frequently performed glaucoma surgery. Still, complications such as leakage and infection are quite common and are even exacerbated by other risk factors and sociodemographic characteristics [12]. Minimally invasive glaucoma surgeries (MIGS) have recently emerged as popular treatments for POAG, with prior studies commending their reduction in the need for pharmacotherapy, surgery time, and the risk of adverse surgical complications [13, 14]. However, their full range of complications has not been fully characterized due to their popularity and varying outcomes across cohort studies [15]. Moreover, their complications in the context of DR have not been extensively studied in the current literature [16]. While MIGS are generally regarded as safer alternatives with fewer post-operative complications compared to traditional glaucoma surgeries, their outcomes in POAG patients with concomitant DR remain understudied [17, 18].

This study aims to bridge this knowledge gap by comparing MIGS outcomes in POAG patients with and without comorbid DR. By focusing on this specific patient cohort, we aim to determine whether DR alters the risk profile associated with MIGS procedures. By analyzing a comprehensive real-world patient dataset, we aim to provide valuable insights into the safety profile of MIGS in this patient population, ultimately informing clinical decision-making and enhancing patient care.

## Methods

### Study design and data source

We conducted a retrospective cohort study using the TriNetX global health network, a federated network of healthcare organizations that provides de-identified patient data for research. This database enables the generation of real-world evidence by analyzing longitudinal clinical data from multiple healthcare organizations.

### Study population

Two groups of adult patients (over 18 years of age) were identified: patients diagnosed with primary open-angle glaucoma (POAG) who underwent minimally invasive glaucoma surgery (MIGS) and patients diagnosed with both POAG and diabetic retinopathy (DR) who underwent MIGS.

### Inclusion and exclusion criteria

Inclusion criteria were based on specific ICD-10 diagnosis codes for POAG (H40.11) and various forms of DR (E08.31-E08.35, E10.31-E10.35, E11.31-E11.35, E13.31-E13.35). MIGS procedures were identified using CPT codes (65820, 66174, 66989, 66999, 0671 T, 0191 T, 0253 T, 66,183, 0376 T), as detailed in Supplementary Table S1. Exclusion criteria included patients with other forms of glaucoma (e.g., secondary glaucoma) and those on systemic corticosteroid medication. The specific exclusion codes are listed in Supplementary Table S2.

### Propensity score matching

Propensity score matching was used to minimize confounding factors and balance baseline characteristics between the two groups. The matching was based on age at index date, race (White, Black or African American, Asian), ethnicity (Hispanic or Latino, Not Hispanic or Latino), sex (Male, Female), and comorbidities, including hypertensive diseases (ICD-10 codes I10-I15), acute kidney failure and chronic kidney disease (ICD-10 codes N17-N19), and cerebral infarction (ICD-10 code I63). The propensity score was calculated using logistic regression, and matching was performed using a nearest-neighbor algorithm with a caliper width of 0.2 standard deviations of the propensity score’s logit.

### Outcome measures

The primary outcomes of interest included vision disturbances and blindness, hypotony, ocular hypertension, choroidal detachment, cataract formation, eye infection, and ocular hemorrhage. These outcomes were identified using specific ICD-10 codes as outlined in Supplementary Table S3. The follow-up period for assessing these outcomes was one year post-MIGS procedure.

### Statistical analysis

Descriptive statistics were used to characterize the study population, including means and standard deviations for continuous variables and frequencies and percentages for categorical variables. Relative risks (RR) with 95% confidence intervals (CI) were calculated for each outcome. A *p*-value < 0.05 was considered statistically significant. To assess the robustness of our findings, we conducted sensitivity analyses stratifying patients by diabetic retinopathy severity. All statistical analyses were performed using built-in analytical tools of TriNetX and R version 4.0.3.

## Results

### Study characteristics

The study initially included 550 patients with DR and 7220 without DR, all of whom underwent minimally invasive glaucoma surgery. Before propensity score matching, significant differences in baseline characteristics were observed between the groups.

Patients with DR were younger (68.0 ± 9.6 years vs. 70.5 ± 9.6 years, *p* < 0.001) and had a different racial composition, with fewer White patients (43.3% vs. 59.9%, *p* < 0.001) and more Black or African American patients (29.3% vs. 18%, *p* < 0.001). The DR group also had a higher proportion of Hispanic or Latino patients (16.3% vs. 9.2%, *p* < 0.001). Comorbidities were most prevalent in the DR group, including hypertensive diseases (73.7% vs. 38.4%, *p* < 0.001), acute and chronic kidney disease (27.6% vs. 7.8%, *p* < 0.001), and cerebral infarction (7.3% vs. 2.4%, *p* < 0.001).

Propensity score matching yielded 518 patients per group, balancing baseline characteristics. Post-matching, there were no statistically significant differences in age, sex, race, ethnicity, or comorbidities between the groups. Table 1 provides a comprehensive overview of the baseline characteristics before and after propensity score matching.

**Table 1:** Characteristics of glaucoma patients before and after propensity score matching analysis

Variables	Before propensity score matching			After propensity score matching		
	DR	No DR	*p*-value	DR	No DR	*p*-value
Number	550	7220		518	518	
Demographics						
Age at Index, years	68.0 ± 9.6	70.5 ± 9.6	**< 0.001**	68.1 ± 9.6	68.9 ± 9.5	0.23
Sex						
Female	264 (49.5%)	3503 (49.8%)	0.897	256 (49.4%)	255 (49.2%)	0.95
Male	256 (48%)	3092 (44%)	0.069	249 (48.1%)	253 (48.8%)	0.80
Race						
White	231 (43.3%)	4211 (59.9%)	**< 0.001**	230 (44.4%)	224 (43.2%)	0.70
Black or African American	156 (29.3%)	1267 (18%)	**< 0.001**	150 (29%)	157 (30.3%)	0.63
Asian	24 (4.5%)	279 (4%)	0.544	23 (4.4%)	27 (5.2%)	0.56
Ethnicity						
Not Hispanic or Latino	318 (59.7%)	4598 (65.4%)	**0.007**	312 (60.2%)	319 (61.6%)	0.65
Hispanic or Latino	87 (16.3%)	646 (9.2%)	**< 0.001**	80 (15.4%)	78 (15.1%)	0.86
Comorbidities						
Hypertensive diseases	393 (73.7%)	2700 (38.4%)	**< 0.001**	378 (73%)	381 (73.6%)	0.83
Acute and chronic kidney disease	147 (27.6%)	545 (7.8%)	**< 0.001**	132 (25.5%)	137 (26.4%)	0.72
Cerebral infarction	39 (7.3%)	170 (2.4%)	**< 0.001**	33 (6.4%)	32 (6.2%)	0.89

Data are presented as mean (standard deviation) or frequency (percentage). Two-sided Chi-squared and Student t-tests were used. Statistically significant results (*p* < 0.05) are shown in bold. DR: diabetic retinopathy

### Outcomes analysis

The mean follow-up time for MIGS patients before matching was 3.68 years (SD = 3.36) for the DR group and 3.71 years (SD = 3.33) for the non-DR group. After matching, the durations were 3.67 years (SD = 3.35) and 3.57 years (SD = 3.38), respectively.

Analysis of post-operative outcomes revealed significant differences between DR and non-DR patients. Vision loss at the end of the follow-up occurred in 32.8% of DR patients compared to 24.4% of non-DR patients (*p* = 0.005). Cataract formation rates at 3 months were 44.4% in DR patients versus 50.6% in non-DR patients (*p* = 0.046), and at 6 months were 53.3% versus 59.5%, respectively (*p* = 0.048). Notably, ocular hemorrhage was observed in 13.4% of DR patients compared to 4.3% of non-DR patients (*p* < 0.001). No statistically significant differences were observed for hypotony, ocular hypertension, or eye infection at any time point (Table 2).

**Table 2:** Comparison of outcomes between patients with and without diabetic retinopathy after minimally invasive glaucoma surgery

Outcome	Time	DR	No DR	*p*-value
Vision loss	3 months	49 (9.5%)	47 (9.1%)	0.83
	6 months	66 (13.2%)	66 (13.2%)	1.0
	Any time	152 (32.8%)	113 (24.4%)	**0.005**
Hypotony	3 months	10 (1.9%)	10 (1.9%)	1.0
	6 months	10 (2%)	10 (2%)	1.0
	Any time	10 (2.2%)	10 (2.2%)	1.0
Ocular hypertension	3 months	27 (5.2%)	26 (5%)	0.88
	6 months	38 (7.6%)	27 (5.4%)	0.15
	Any time	69 (14.9%)	56 (12.1%)	0.21
Cataract formation	3 months	230 (44.4%)	262 (50.6%)	**0.046**
	6 months	267 (53.3%)	298 (59.5%)	**0.048**
	Any time	360 (77.6%)	368 (79.3%)	0.52
Eye infection	3 months	13 (2.5%)	10 (1.9%)	0.52
	6 months	24 (4.8%)	23 (4.6%)	0.88
	Any time	75 (16.2%)	66 (14.2%)	0.41
Ocular hemorrhage	3 months	13 (2.5%)	10 (1.9%)	0.52
	6 months	19 (3.8%)	10 (2%)	0.09
	Any time	62 (13.4%)	20 (4.3%)	**< 0.001**

Data are presented as frequency (percentage). A two-sided Chi-Square was used. The outcomes are assessed at 3 months, 6 months, and at any time during the follow-up period. Statistically significant results (*p* < 0.05) are shown in bold. DR: diabetic retinopathy

**Fig. 1:**
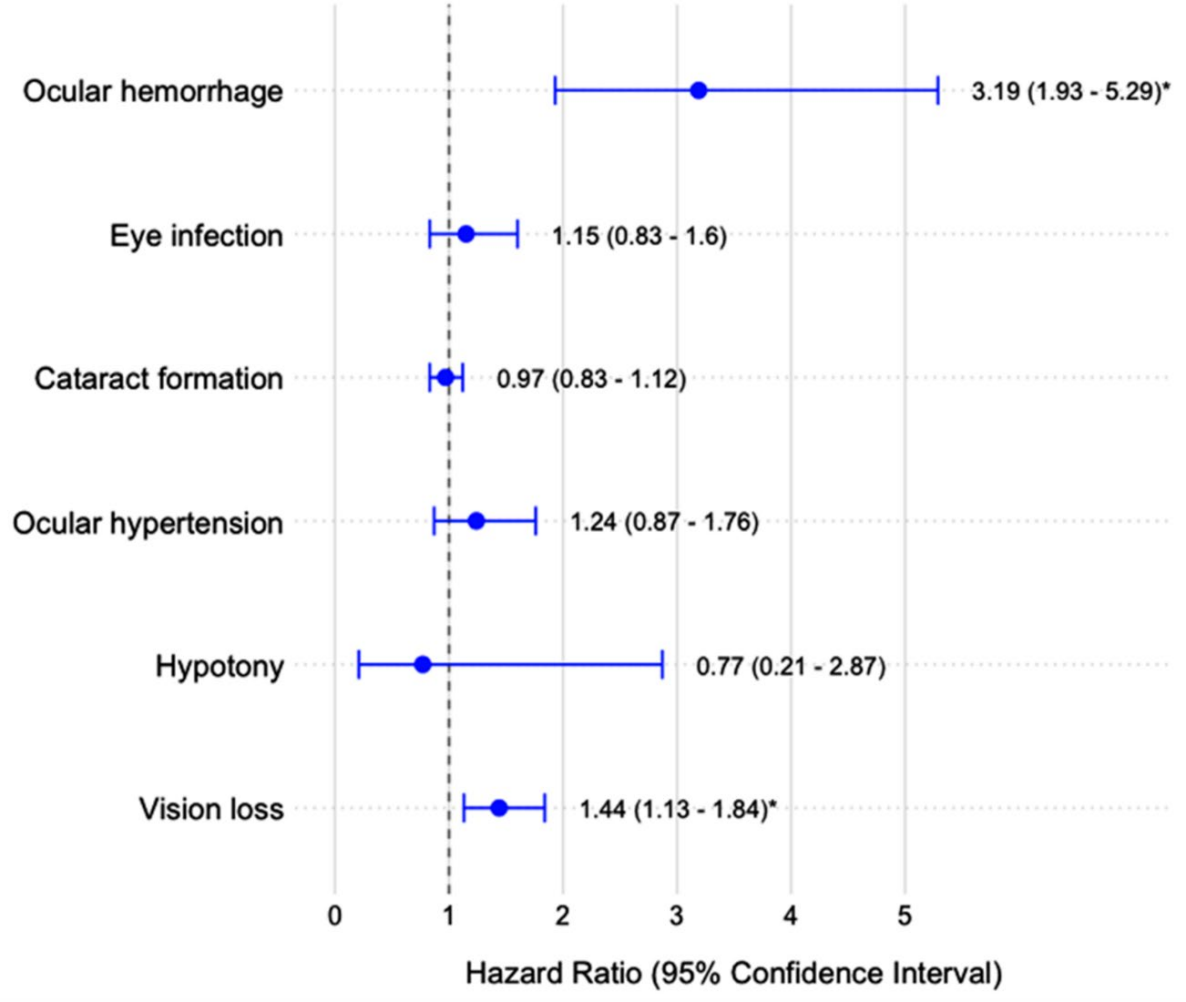
Risk for poor outcomes following minimally invasive glaucoma surgery. The hazard ratios (HRs) are represented by dots, with horizontal lines indicating the 95% confidence intervals for outcomes compared with patients with diabetic retinopathy and those without. Hazard ratios greater than 1 indicate an increased risk for patients with diabetic retinopathy, while those less than 1 indicate a decreased risk. Statistically significant results (where the confidence interval does not include 1) are shown with a*

Cox regression analysis (Fig. 1) indicated that DR patients had a 44.3% higher risk of vision loss (HR 1.443, 95% CI 1.13–1.841) and a three-fold higher risk of ocular hemorrhage (HR 3.194, 95% CI 1.929–5.288) compared to non-DR patients. Other outcomes, including hypotony, ocular hypertension, cataract formation, and eye infection, showed no statistically significant differences in risk between the two groups.

## Discussion

This large-scale retrospective cohort study provides important insights into the complications of MIGS in patients with and without DR. This study encompasses patients from several nations and those aged 18 years or older. It is well known that older age (especially 40 years or older) is a significant risk factor for POAG, although younger patients may also be vulnerable in the presence of certain genetic mutations [19, 20]. Nevertheless, our findings suggest that patients with DR undergoing MIGS face increased risks of certain post-operative complications and may have a compounding effect on visual outcomes.

This association can be due to several factors. First, the underlying vascular fragility and tortuosity in patients with DR can lead to weak vasculature that can be further harmed during surgery and contribute to vision loss [21]. Indeed, such vulnerable ocular vessels in DR can be due to chronic hyperglycemia, which damages the endothelium, as well as elevated levels of VEGF in the aqueous humor, which promote neovascularization of further weakened vessels [22, 23]. Second, the compromised retinal function in DR patients can be due to oxidative stress, loss of blood-retinal barrier integrity, and inflammation that could be further impacted by undergoing MIGS [24]. Third, there is a delayed wound-healing process in DR due to the damaged endothelium, which can lead to imbalances in secreted anti-fibrinolytic substances, such as von Willebrand factor and plasminogen activator inhibitor-1, and pro-fibrinolytic substances, such as tissue plasminogen activator [25]. The impaired ability to heal wounds could prevent patients from healing properly from the tissue trauma that can arise from undergoing any procedural intervention.

The significantly higher incidence of vision loss in DR patients (32.8% vs. 24.4%, *p* = 0.005) is a key finding, with DR causing a 44.3% increased risk (HR 1.443, 95% CI 1.13–1.841). Interestingly, several studies support this. Liu et al. examined visual acuity after the Ahmed tube shunt procedure. They noted that one patient who had chronic, uncontrolled DR had a tractional and rhegmatogenous retinal detachment, which led to extensive vision loss within six months after surgery [26]. One possible explanation for this is the ocular hypoperfusion induced during MIGS. Although the literature shows some discrepancies among types of MIGS, a plethora of studies have shown that various MIGS can significantly decrease intraocular pressure and the subsequent need for pressure-lowering drops [27, 28]. Sudden, modest reductions in intraocular pressure can cause retinal ischemia, which may contribute to vision loss [29, 30]. In the current era, instrument sizes for typically extensive surgeries, such as vitrectomies, have become smaller to enable minimally invasive procedures. This allows for more controlled intraocular pressure drops during surgery, potentially mediating the risk of hypoperfusion.

The threefold increase in the risk of any ocular hemorrhage among DR patients (HR 3.194, 95% CI 1.929–5.288) is a strong finding that may also have contributed to the higher risk of vision loss in this patient cohort. This is not surprising given that post-operative hemorrhagic events in general occur in the majority of cases, depending on the specific procedure [31]. We describe ocular hemorrhage as either retinal, conjunctival, vitreous, or choroidal. Although hyphema, or anterior chamber hemorrhage, is a common type of ocular hemorrhage after MIGS, we did not include it as a study outcome. It can be difficult to distinguish post-operative hyphema with or without concomitant clot formation, especially among patients with DR who are more vulnerable to such neovascular complications than those without DR. Indeed, those with clot formation may experience greater post-operative intraocular pressures and higher reoperation rates, prolonging resolution times [32]. Unfortunately, we were unable to assess the incidence of clot formation in the DR and non-DR cohorts, which may limit the utility of assessing hyphema incidence. This is indeed a limitation of a retrospective cohort study utilizing an administrative database, and thus requires future trials to assess the incidence and resolution periods of hyphema with and without clot formation.

Interestingly, our study found lower rates of cataract formation in DR patients at 3 and 6 months post-surgery. One potential explanation lies in the underlying pathophysiology: patients with DR may have aqueous humor with greater antioxidant activity, which may suppress post-operative inflammatory cascades that contribute to early lens opacification [33]. Indeed, given that these were short-term findings, our follow-up window may preferentially under-detect cataract formation in the DR cohort and should be interpreted with caution. Moreover, clinicians may adopt more conservative perioperative steroid prescribing practices in diabetic patients due to a known risk of increased intraocular pressure, thereby mitigating a contributing factor to early cataract formation [34].

The lack of significant differences in rates of hypotony, ocular hypertension, and eye infection between the groups is reassuring, suggesting that MIGS can be performed in DR patients without substantially increasing the risk of these specific complications. This may indicate that the surgical technique itself is equally safe in both groups, but the unique vascular and structural characteristics of DR eyes lead to specific complications.

Our study has several strengths, including its large sample size, the use of propensity score matching to control for confounding, and the assessment of multiple outcomes across different time points. However, it also has limitations inherent to retrospective administrative datasets, such as potential coding errors and a lack of detailed clinical information. This becomes clinically relevant when describing the context of the procedures, such as whether they were isolated or performed in addition to phacoemulsification. Furthermore, we were unable to stratify complications by MIGS type, which may be relevant to clinicians when deciding on specific instruments or treatments. Moreover, we were unable to compare complication risks based on lab value-based DM control.

To our knowledge, this is the first large-scale retrospective cohort study to demonstrate that DR can increase the risk of complications after MIGS, despite its known benefits. This suggests two recommendations. First, controlling DM before any MIGS may reduce this risk. Second, further studies are needed to evaluate the risk of these complications by MIGS type.

## Conclusions

MIGS appears to be a viable option for glaucoma management in patients with DR, but it carries increased risks of vision loss and ocular hemorrhage in this population. These findings should inform clinical decision-making, surgical planning, and post-operative care strategies for DR patients undergoing MIGS. We recommend enhanced pre-operative counseling, consideration of modified surgical techniques, more intensive post-operative monitoring, and further research into the mechanisms underlying these increased risks. Future studies should also investigate whether certain subgroups of DR patients have differential risks, thereby further refining patient selection and management strategies for MIGS in this population.

## Supplementary Information

Below is the link to the electronic supplementary material.

Supplementary file1 (PDF 141 KB)

## Data Availability

The data used in this study are not publicly accessible due to licensing and contractual agreements with the TriNetX research network. Access to these data requires an institutional subscription and formal approval from TriNetX. Researchers interested in replicating or extending this work may request access to the TriNetX platform (https://trinetx.com/) through their affiliated institutions, subject to TriNetX approval and compliance with its data use policies. The specific query parameters used in our analysis can be provided upon reasonable request to the corresponding author.
