# Supplementary material for: Complications of minimally invasive surgery for primary open-angle glaucoma in patients with diabetic retinopathy: a retrospective cohort study
Source: Int Ophthalmol. 2026 Feb 5;46(1):103. doi: 10.1007/s10792-026-03945-8 (PMC12876447; doi:10.1007/s10792-026-03945-8)
Supplement: Supplementary file 1 — Supplementary file1 (PDF 141 KB) [file 10792_2026_3945_MOESM1_ESM.pdf]

Complications of Minimally Invasive Surgery for Primary Open-Angle Glaucoma in Patients with Diabetic Retinopathy: A Retrospective Cohort Study

Table S1. Inclusion Criteria.

Criteria	Category	Code	Description
Open Angle Glaucoma	Diagnosis	UMLS:ICD10CM:H40.11	Primary open-angle glaucoma
Diabetic Retinopathy	Diagnosis	UMLS:ICD10CM:E08.31	Diabetes mellitus due to underlying condition with unspecified diabetic retinopathy
	Diagnosis	UMLS:ICD10CM:E08.32	Diabetes mellitus due to underlying condition with mild nonproliferative diabetic retinopathy
	Diagnosis	UMLS:ICD10CM:E08.33	Diabetes mellitus due to underlying condition with moderate nonproliferative diabetic retinopathy
	Diagnosis	UMLS:ICD10CM:E08.34	Diabetes mellitus due to underlying condition with severe nonproliferative diabetic retinopathy
	Diagnosis	UMLS:ICD10CM:E08.35	Diabetes mellitus due to underlying condition with proliferative diabetic retinopathy
	Diagnosis	UMLS:ICD10CM:E10.31	Type 1 diabetes mellitus with unspecified diabetic retinopathy
	Diagnosis	UMLS:ICD10CM:E10.32	Type 1 diabetes mellitus with mild nonproliferative diabetic retinopathy
	Diagnosis	UMLS:ICD10CM:E10.33	Type 1 diabetes mellitus with moderate nonproliferative diabetic retinopathy
	Diagnosis	UMLS:ICD10CM:E10.34	Type 1 diabetes mellitus with severe nonproliferative diabetic retinopathy
	Diagnosis	UMLS:ICD10CM:E10.35	Type 1 diabetes mellitus with proliferative diabetic retinopathy
	Diagnosis	UMLS:ICD10CM:E11.31	Type 2 diabetes mellitus with unspecified diabetic retinopathy
	Diagnosis	UMLS:ICD10CM:E11.32	Type 2 diabetes mellitus with mild nonproliferative diabetic retinopathy
	Diagnosis	UMLS:ICD10CM:E11.33	Type 2 diabetes mellitus with moderate nonproliferative diabetic retinopathy
	Diagnosis	UMLS:ICD10CM:E11.34	Type 2 diabetes mellitus with severe nonproliferative diabetic retinopathy
	Diagnosis	UMLS:ICD10CM:E11.35	Type 2 diabetes mellitus with proliferative diabetic retinopathy
	Diagnosis	UMLS:ICD10CM:E13.31	Other specified diabetes mellitus with unspecified diabetic retinopathy
	Diagnosis	UMLS:ICD10CM:E13.32	Other specified diabetes mellitus with mild nonproliferative diabetic retinopathy
	Diagnosis	UMLS:ICD10CM:E13.33	Other specified diabetes mellitus with moderate nonproliferative diabetic retinopathy
	Diagnosis	UMLS:ICD10CM:E13.34	Other specified diabetes mellitus with severe nonproliferative diabetic retinopathy
	Diagnosis	UMLS:ICD10CM:E13.35	Other specified diabetes mellitus with proliferative diabetic retinopathy
	Procedure	UMLS:CPT:65820	Goniotomy

Minimally Invasive Glaucoma Surgery	Procedure	UMLS:CPT:66174	Transluminal dilation of aqueous outflow canal (eg, canaloplasty); without retention of device or stent
	Procedure	UMLS:CPT:66989	Extracapsular cataract removal with insertion of intraocular lens prosthesis (1-stage procedure), manual or mechanical technique (eg, irrigation and aspiration or phacoemulsification), complex, requiring devices or techniques not generally used in routine cataract surgery (eg, iris expansion device, suture support for intraocular lens, or primary posterior capsulorrhexis) or performed on patients in the amblyogenic developmental stage; with insertion of intraocular (eg, trabecular meshwork, supraciliary, suprachoroidal) anterior segment aqueous drainage device, without extraocular reservoir, internal approach, one or more
	Procedure	UMLS:CPT:66999	Unlisted procedure, anterior segment of eye
	Procedure	UMLS:CPT:0671T	Insertion of anterior segment aqueous drainage device into the trabecular meshwork, without external reservoir, and without concomitant cataract removal, one or more
	Procedure	UMLS:CPT:0191T	Insertion of anterior segment aqueous drainage device, without extraocular reservoir, internal approach, into the trabecular meshwork; initial insertion (deprecated 2022)
	Procedure	UMLS:CPT:0253T	Insertion of anterior segment aqueous drainage device, without extraocular reservoir, internal approach, into the suprachoroidal space
	Procedure	UMLS:CPT:66183	Insertion of anterior segment aqueous drainage device, without extraocular reservoir, external approach
	Procedure	UMLS:CPT:0376T	Insertion of anterior segment aqueous drainage device, without extraocular reservoir, internal approach, into the trabecular meshwork; each additional device insertion (List separately in addition to code for primary procedure) (deprecated 2022)

Table S2. Exclusion Criteria.

Criteria	Category	Code	Description
Other Eye Diseases	Diagnosis	UMLS:ICD9CM:365.4	Glaucoma associated with congenital anomalies, dystrophies, and systemic syndromes
	Diagnosis	UMLS:ICD10CM:H40.3	Glaucoma secondary to eye trauma
	Diagnosis	UMLS:ICD10CM:H40.4	Glaucoma secondary to eye inflammation
	Diagnosis	UMLS:ICD10CM:H40.5	Glaucoma secondary to other eye disorders
	Diagnosis	UMLS:ICD10CM:H40.6	Glaucoma secondary to drugs
	Diagnosis	UMLS:ICD10CM:H40.8	Other glaucoma
	Diagnosis	UMLS:ICD10CM:H40.9	Unspecified glaucoma
	Diagnosis	UMLS:ICD9CM:365.6	Glaucoma associated with other ocular disorders
	Diagnosis	UMLS:ICD10CM:H40.30	Glaucoma secondary to eye trauma, unspecified eye
	Diagnosis	UMLS:ICD10CM:H40.32	Glaucoma secondary to eye trauma, left eye
	Diagnosis	UMLS:ICD10CM:H40.31	Glaucoma secondary to eye trauma, right eye
	Diagnosis	UMLS:ICD10CM:H40.33	Glaucoma secondary to eye trauma, bilateral
Corticosteroid Medication	Medication	NLM:ATC:H02	Corticosteroids for systematic use
	Medication	NLM:ATC:S03B	Corticosteroids

Table S3. Outcome measures.

Criteria	Category	Code	Description
Ocular hypertension	Diagnosis	UMLS:ICD10CM:H40.05	Ocular hypertension
Hypotony	Diagnosis	UMLS:ICD10CM:H44.4	Hypotony of eye.
Visual disturbances and blindness	Diagnosis	UMLS:ICD10CM:H53	Visual disturbances
	Diagnosis	UMLS:ICD10CM:H54	Blindness and low vision
Choroidal detachment	Diagnosis	UMLS:ICD10CM:H31.4	Choroidal detachment
Cataract formation	Diagnosis	UMLS:ICD10CM:H25	Age-related cataract
	Diagnosis	UMLS:ICD10CM:H26	Other cataract
	Diagnosis	UMLS:SNOMED:110473004	Cataract surgery
	Diagnosis	UMLS:ICD10CM:Z98.41	Cataract extraction status, right eye
	Diagnosis	UMLS:ICD10CM:Z98.49	Cataract extraction status, unspecified eye
	Diagnosis	UMLS:ICD10CM:Z98.42	Cataract extraction status, left eye
	Diagnosis	UMLS:ICD10CM:H28	Cataract in diseases classified elsewhere
Eye infection	Diagnosis	UMLS:ICD10CM:H01	Other inflammation of eyelid
	Diagnosis	UMLS:ICD10CM:H10	Conjunctivitis
	Diagnosis	UMLS:ICD10CM:H16	Keratitis
	Diagnosis	UMLS:ICD10CM:H15.0	Scleritis
	Diagnosis	UMLS:ICD10CM:H15.1	Episcleritis
	Diagnosis	UMLS:ICD10CM:H20.0	Acute and subacute iridocyclitis
	Diagnosis	UMLS:ICD10CM:H20.2	Lens-induced iridocyclitis
	Diagnosis	UMLS:ICD10CM:H20.8	Other iridocyclitis
	Diagnosis	UMLS:ICD10CM:H20.9	Unspecified iridocyclitis
Ocular hemorrhage	Diagnosis	UMLS:ICD10CM:H35.6	Retinal hemorrhage
	Diagnosis	UMLS:ICD10CM:H11.3	Conjunctival hemorrhage

	Diagnosis	UMLS:ICD10CM:H43.1	Vitreous hemorrhage
	Diagnosis	UMLS:ICD10CM:H31.3	Choroidal hemorrhage and rupture
